# The advance of assembly of exopolysaccharide Psl biosynthesis machinery in *Pseudomonas aeruginosa*

**DOI:** 10.1002/mbo3.857

**Published:** 2019-05-08

**Authors:** Huijun Wu, Di Wang, Maomao Tang, Luyan Z. Ma

**Affiliations:** ^1^ State Key Laboratory of Microbial Resources Institute of Microbiology, Chinese Academy of Sciences Beijing China; ^2^ University of Chinese Academy of Sciences Beijing China

**Keywords:** biofilm, exopolysaccharide Psl, glycosyl hydrolase, *Pseudomonas aeruginosa*

## Abstract

Biofilms are microbial communities embedded in extracellular matrix. Exopolysaccharide Psl (ePsl) is a key biofilm matrix component that initiates attachment, maintains biofilms architecture, and protects bacteria within biofilms of* Pseudomonas aeruginosa*, an opportunistic pathogen. There are at least 12 Psl proteins involved in the biosynthesis of this exopolysaccharide. However, it remains unclear about the function of each Psl protein and how these proteins work together during the biosynthesis of ePsl. PslG has been characterized as a degrader of ePsl in extracellular or periplasm and PslD is predicted to be a transporter. In this study, we found that PslG and its glycoside hydrolytic activity were also involved in the biosynthesis of ePsl. PslG localized mainly in the inner membrane and some in the periplasm. The inner membrane association of PslG was critical for the biosynthesis of ePsl. The expression of PslA, PslD, and PslE helped PslG remain in the inner membrane. The bacterial two‐hybrid results suggested that PslE could interacted with either PslA, PslD, or PslG. The strongest interaction was found between PslE and PslD. Consistently, PslD was disabled to localize on the outer membrane in the Δ*pslE* strain, suggesting that the PslE‐PslD interaction affected the localization of PslD. Our results shed light on the assembly of ePsl biosynthesis machinery and suggested that the membrane‐associated PslG was a part of ePsl biosynthesis proteins complex.

## Graphical abstract

In this study, we shed light on the assembly of exopolysaccharide Psl (ePsl) biosynthesis machinery in *Pseudomonas aeruginosa*. We improve our understanding about the function of PslG, the interactions among Psl proteins and the effect of proteins interactions on the localization of Psl protein during ePsl biosynthesis .

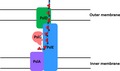

## 1 INTRODUCTION

Structured, surfaced‐associated communities of bacteria known as biofilms are prevalent in nature, industrial, and clinical settings (Costerton, Lewandowski, Caldwell, Korber, & Lappin‐Scott, 1995; Stoodley, Sauer, Davies, & Costerton, 2002). Biofilm matrix, which plays a key role in biofilm development, is extracellular substance secreted by biofilm bacteria. Although the component of biofilm matrix differs among species, it is generally composed of polysaccharides, proteins, and nucleic acids (Flemming & Wingender, 2010; Stoodley et al., 2002). The extracellular polysaccharides have a key role in biofilm matrix function because they promote attachment to surfaces and other cells, act as a scaffold to help maintain biofilm structure, and protect cells from antibiotics and host defenses (Häussler & Parsek, 2010; Stewart & Costerton, 2001; Stewart & Costerton, 2001). Although the importance of exopolysaccharide is widely accepted, the exact mechanism underlying their biosynthesis remains poorly understood. A better understanding of the molecular mechanisms of polysaccharide biosynthesis may provide strategies for the control of chronic infections and problems related to biofilm formation.

*Pseudomonas aeruginosa* is an opportunistic human pathogen that can cause life‐threatening infections in cystic fibrosis patients and individuals with compromised immune system (Govan & Deretic, 1996; Lyczak, Cannon, & Pier, 2000; Ramsey & Wozniak, 2005). *P. aeruginosa* is a model organism to study the process of biofilm development. There are at least three unique exopolysaccharides implicated in *P. aeruginosa* biofilm development, alginate, ePsl, and Pel (Branda, Vik, Friedman, & Kolter, 2005; Colvin et al., 2012; Ma, Jackson, Landry, Parsek, & Wozniak, 2006; Ramsey & Wozniak, 2005). Alginates are anionic exopolysaccharides composed of variable proportions of 1,4‐linked β‐ᴅ‐mannuronic acid and its C‐5 epimer α‐ʟ‐guluronic acid (Hay, Rehman, Ghafoor, & Rehm, 2010). Twelve proteins are required for the biosynthesis of alginate (Chitnis & Ohman, 1993; Franklin, Nivens, Weadge, & Howell, 2011). They have been characterized to elucidate the alginate biosynthetic mechanism, including polymerization, epimerization, acetylation, secretion, and regulation (Franklin et al., 2011; Moradali, Donati, Sims, Ghods, & Rehm, 2015; Rehman, Wang, Moradali, Hay, & Rehm, 2013). Pel is a positively charged polysaccharide composed of partially acetylated 1–4 glycosidic linkages of *N*‐acetylgalactosamine and *N*‐acetylglucosamine (Jennings et al., 2015). A seven‐gene operon (*pelABCDEFG*) is essential for Pel biosynthesis (Friedman & Kolter, 2004; Vasseur, Vallet‐Gely, Soscia, Genin, & Filloux, 2005). Structural and biochemical analyses have shed light on the understanding of Pel polymerization, deacetylation, and exportation (Colvin et al., 2013; Ghafoor, Jordens, & Rehm, 2013; Marmont et al., 2017; Whitney et al., 2012).

The ePsl is a neutral pentasaccharide repeat containing ᴅ‐mannose, ᴅ‐glucose, and ʟ‐rhamnose (Byrd et al., 2009). The polysaccharide synthesis locus (*psl*) contains 15 genes, 11 of which (*pslACDEFGHIJKL*) are required for ePsl biosynthesis (Byrd et al., 2009). However, the function of each Psl protein remains largely unknown. It has been reported that PslB is a bifunctional enzyme and is involved in sugar‐nucleotide precursor production for ePsl biosynthesis (Byrd et al., 2009; Lee, Chang, Venkatesan, & Peng, 2008). PslD is a secreted protein and may play a role in exopolysaccharide export (Campisano, Schroeder, Schemionek, Overhage, & Rehm, 2006). Our previous study (Yu et al., 2015) has demonstrated that PslG is an endoglycosidase mainly targeted ePsl and, the catalytic residues E165 and E276 are critical for the hydrolytic activity. PslG can degrade ePsl to prevent biofilm formation and disassemble existing biofilm when supplied exogenously. While whether PslG is involved in the biosynthesis of ePsl remains controversial. Byrd et al. (2009) considered PslG was required for the biosynthesis of ePsl. On the contrary, Baker et al. (2015) found that neither PslG nor its enzymatic activity appeared to be required for ePsl biosynthesis and biofilm formation. Strain PAO1Δ*pslG* constructed by Byrd et al. (2009) has deleted a cis‐acting element located in the 3’ of *pslG* that altered the translation of *pslH* (Baker et al., 2015), while, the Δ*pslG* strain constructed by Baker et al. (2015) is in the background of a *psl* overexpression strain PAO1Δ*pelF*P_BAD_ *psl* rather than wild type PAO1.

Bioinformatic analyses suggest that ePsl biosynthesis mechanism resembles the biosynthesis of *Escherichia coli* group 1 capsular polysaccharides, with PslA, PslD, and PslE similar to WbaP, Wza, and Wzc, respectively (Franklin et al., 2011). It is proposed that biosynthesis and translocation of ePsl is temporally and spatially coupled by multiprotein complex. Nevertheless, there has not been any investigation about the interaction and localization of Psl proteins that involved in the ePsl biosynthesis.

In this study, we further investigate the role of PslG and its hydrolytic activity on the biosynthesis of ePsl in *P. aeruginosa* PAO1. Interactions among Psl proteins (PslA, PslD, PslG, and PslE) and their effects on the subcellular localization of Psl proteins have been examined. Our results shed light on the assembly of ePsl biosynthesis machinery.

## 2 MATERIALS AND METHODS

### 2.1 Bacterial strains and growth conditions

Bacterial strains and plasmids used in this study are listed in Table 1. Unless indicated, *E. coli* strains were grown at 37°C in Luria Bertani Broth (LB, Becton Dickinson), *P. aeruginosa* stains at 37°C in LB without sodium chloride (LBNS) or Jensen's, a chemically defined medium (Jensen, Fecycz, & Campbell, 1980). L‐arabinose (Sigma) was used as inducer for genes transcribed from P_BAD_ promoter in *P. aeruginosa*. Antibiotics for *P. aeruginosa* were added at the following concentrations: gentamicin 30 μg/ml; ampicillin 100 μg/ml; carbenicillin 300 μg/ml; chloramphenicol 25 μg/ml; tetracycline 12.5 μg/ml. Gentamicin at 15 μg/ml was used for *E. coli*. For *Pseudomonas* selection media, Irgasan at 25 μg/ml was used.

**Table 1:** The strains and plasmids used in this study

Strain or plasmid	Genotype and/or relevant characteristics	Source or reference
*P. aeruginosa* PAO1 series strains		
*P. aeruginosa* PAO1	Prototroph	Holloway (1955)
Δ*pslG2*	In‐frame deletion of *pslG*	This study
Δ*pslA*	In‐frame deletion of *pslA*	Byrd et al. (2009)
Δ*pslD*	In‐frame deletion of *pslD*	Byrd et al. (2009)
Δ*pslE*	In‐frame deletion of *pslE*	Byrd et al. (2009)
WFPA800	ePsl‐negative strain, *psl* operon promoter deletion mutant, ΔP*_psl_*	Ma et al. (2006)
WFPA801	ePsl‐overproduced strain, P_BAD_‐*psl*	Ma et al. (2006)
WFPA801Δ*pslA*	In‐frame deletion of *pslA*	This study
WFPA801Δ*pslD*	In‐frame deletion of *pslD*	This study
WFPA801Δ*pslE*	In‐frame deletion of *pslE*	This study
Δ*pslG2*::*pslG_E165Q + E276Q_*	*pslG* was replaced by the active site mutated *pslG* (E165Q + E276Q)	This study
Δ*pslG2*::*pslG*	*pslG* was knocked into the *pslG* deletion mutant	This study
*E.coli* strains		
XL1‐Blue MRF’ kan	Reporter strain of BacterioMatch II Two‐Hybrid System	Zhang et al. (2009)
BL21(DE3)	F‐ *ompT gal [dcm] [lon] hsdS_B_* (r_B_‐m_B_‐; an *E. coli* B strain) with DE3, a λ prophage carrying T7 RNA polymerase gene	Novagen
Plasmids		
pHERD20T	*E. coli‐P. aeruginosa* shuttle plasmid containing arabinose inducible P_BAD_ promoter, Ap^r^	Qiu, Damron, Mima, Schweizer, and Yu (2008)
pG	pHERD20T with *pslG*, Ap^r^	Yu et al. (2015)
pGDM	pHERD20T with active sites mutated *pslG* (E165Q + E276Q), Ap^r^	This study
pBT	Bait vector of BacterioMatch II Two‐Hybrid System, Cm^r^	Zhang et al. (2009)
pTRG	Target vector of BacterioMatch II Two‐Hybrid System, Tc^r^	Zhang et al. (2009)
pEX18Gm	Cloning vector, Gm^r^	Hoang, Karkhoff‐Schweizer, Kutchma, and Schweizer (1998)
pMA9	pEX18Gm derived plasmid for replacing *psl* operon promoter with *araC*‐p*_BAD_*, Gm^r^	Ma et al. (2006)
pEX‐Δ*pslG2*	pEX18Gm derived plasmid for *pslG* in‐frame deletion, Gm^r^	This study
pEX*‐pslG*	pEX18Gm derived plasmid for knocking in *pslG* into Δ*pslG2*, Gm^r^	This study
pEX*‐pslG_E165Q + E276Q_*	pEX18Gm derived plasmid for replacing *pslG* with *pslG_E165Q + E276Q_*, Gm^r^	This study
pGLO1‐*pslG*	pGLO1 derived plasmid for PslG_31−442 _purification, Ap^r^	Yu et al. (2015)
pSadC‐GFP	C‐terminal Gfp‐tagged SadC expressed in pHERD20T, Ap^r^	Zhu et al. (2016)

### 2.2 Strain construction

The in‐frame *pslG* deletion mutant Δ*pslG2* was constructed by an unmarked, nonpolar deletion strategy as previously described (Carter, Chen, & Lory, 2010). The native sequence located 17 bp upstream of the *pslH* start codon and the 24 bp downstream of the *pslG* start codon was retained. Flanking regions of *pslG* were obtained by overlapping PCR with primers UpPslG2‐F (CCG*GAATTC*CCTCTACCAGTTGAAGGCAC, italics denote the restriction enzyme sites), UpPslG2‐R (TTCACTCCCACAGATAGAGTCCCTTAC), and DwPslG2‐F (ACTCTATCTGTGGGAGTGAAGCCACC), DwPslG2‐R (CCC*AAGCTT*CGACGTTGTGCTCGGTGAG) and then cloned into suicide vector pEX18Gm at EcoRI and HindIII sites, generating plasmid pEX‐Δ*pslG2*. This plasmid was transformed into S17‐1 and subsequently transferred to *P. aeruginosa* by conjugation. For single recombination mutant selection, LBNS plates with 30 μg/ml gentamycin and 25 μg/ml irgasan were used; for double recombination mutant selection, LBNS plates containing 10% sucrose were used. The chromosomal point mutation strain Δ*pslG2*::*pslG_E165Q + E276Q_* was constructed with the similar method described above by using the allelic exchange plasmid pEX‐*pslG_E165Q + E276Q_* to knock in *pslG_E165Q + E276Q_* into Δ*pslG2*. The *psl*‐inducible strains WFPA801*∆pslA*, WFPA801*∆pslD,* and WFPA801*∆pslE* were constructed in accordance with WFPA801 (Ma et al., 2006). Briefly, plasmid pMA9 was transferred into deletion mutants ∆*pslA*, ∆*pslD,* and ∆*pslE*, respectively, and double‐crossover recombinants were selected.

### 2.3 Microtiter dish biofilm assay

In the biofilm attachment assay, 1/100 dilution of a saturated (overnight) culture in Jensen's media for *P. aeruginosa* was inoculated into glass tubes. When the OD_600_ reached 0.5, the culture was inoculated into 96‐well PVC microtiter dish (BD Falcon), and incubated at 30°C for 30 min. Then the planktonic and loosely adherent bacteria cells were washed off by rinsing the plate in water. The remaining surface‐attached cells were stained by 0.1% crystal violet, solubilized in 30% acetic acid, and finally measured (OD_560_) as described previously (Ma et al., 2006; O'Toole, 2011).

### 2.4 Antibody preparation

Anti‐PslG serum was made by Abmart company (Shanghai, China) by using purified PslG_31‐442_ and a 70 d standard protocol. The antiserum against PslG_31‐442 _was absorbed by using *P. aeruginosa* Δ*pslG2* whole cell lysates. The absorption was performed at 4°C for 2 hr by mixing 2 μl anti‐PslG antisera, 60 μl Δ*pslG2* cell lysate in 440 μl of PBST (140 mM NaCl, 2 mM KCl, 8 mM Na_2_HPO_4_, 1.5 mM KH_2_PO_4_, 0.005% Tween) containing 2% BSA and 10 mM EDTA, then centrifuged at 10,000 rpm for 15 min at 4°C. The supernatant was collected as the purified antiserum. Anti‐PslD antibody was made by epitope approach. A synthetic polypeptide (RRVALMREDSEG) corresponding to residues 174–185 of PslD was selected on the basis of an antigenic epitope analysis. The polypeptide was used to immunize rabbits to obtain the polyclonal antibody serum by Abmart (Shanghai, China).

### 2.5 ePsl immuno‐dot blotting and cell extract western blotting analysis

*P. aeruginosa* cell surface associated polysaccharide extracts were obtained from culture that equivalents approximately 4 OD_600_, and examined by immunoblotting using anti‐ePsl antiserum as previously described (Byrd et al., 2009). To induce the transcription of the *pslG* in the recombinant plasmid, arabinose was added to Jensen's media. The immunoblotting data were analyzed using Image Lab software.

Two milliliters of overnight culture (OD_600_ of ~2) grown in LBNS was harvested and resuspended in 100 μl Lysis buffer (50 mM potassium phosphate pH 7.8, 400 mM NaCl, 100 mM KCl, 10% glycerol, 0.5% Triton X‐100, 10 mM imidazole). Samples were frozen in liquid nitrogen and then thawed at 42°C, repeated 3 times to obtain the whole cell extracts. The equivalent amount of whole cell extracts was mixed with 2 × SDS‐PAGE sample buffer and boiled for 5 min. Proteins were separated by SDS‐PAGE and transferred onto polyvinylidene difluoride (PVDF) membrane. The PslG or PslD protein was detected by incubating the membrane with primary antibody against the absorbed anti‐PslG antibody and the anti‐PslD antibody, respectively. RNA polymerase was detected using anti‐RNAp antibody (Abcam, shanghai China). The software Image Lab was used to analyze the immune‐blotting data.

### 2.6 Subcellular fractionation

Subcellular fractionation was adapted from a previously described procedure (Baker et al., 2015; Colvin et al., 2013; Liu & Walsh, 1990; Russell et al., 2011). Briefly, 1 L of *P. aeruginosa* culture grown overnight was harvested by centrifugation (5,000 rpm*,* 30 min, 4°C). The pellet was resuspended in 5 ml buffer I (0.2 M Tris‐HCl pH 8.0, 1 M sucrose, 1 mM EDTA, 1 mg/ml lysozyme) and incubated at room temperature for 5 min. Then 20 ml of ddH_2_O was gently added. The sample was placed on ice for 20 min, and then centrifuged at 45,000 rpm for 45 min at 4°C. The supernatant fraction was collected as periplasmic sample. The pellet was resuspended in 50 ml buffer II (10 mM Tris‐HCl pH 7.5, 5 mM EDTA, 1 mM DTT, 10 μg/ml DNase I), and then applied to sonication. Unlysed cells were removed by centrifugation (16,000 rpm, 20 min, 4°C). The supernatant was further centrifuged at 45,000 rpm for 2 hr at 4°C. The supernatant consisted of the cytoplasmic fraction, and the pellet contained the membrane fraction. The pellet was resuspended in 25 ml buffer III (50 mM Tris‐HCl pH 8.0, 2% (v/v) Triton X‐100, 10 mM MgCl_2_). The sample was centrifuged (35,000 rpm, 30 min, 4°C) and the resulting supernatant contained the inner membrane fraction while the pellet contained the outer membrane fraction. The pellet was washed in 50 ml buffer III twice, and centrifuged at 35,000 rpm for 30 min at 4°C. The samples were dissolved in SDS‐PAGE loading buffer and detected by western blotting using purified anti‐PslG antibody, anti‐PslD antibody, or anti‐Gfp antibody (Abcam, Shanghai China).

### 2.7 Protein expression and purification

PslG_31‐442_ was expressed and purified as previously described (Yu et al., 2015). The first 30 residues of PslG were truncated because they were predicted to be a signal peptide by the Signal P4.1 server. Briefly, *E. coli* BL21 (DE3) carried pGLO1‐*pslG* was grown in 1 L LB containing 100 μg/ml ampicillin at 37°C. When the OD_600_ of the culture reached 0.5–0.8, protein expression was induced overnight with 0.1 mM isopropyl β‐D‐thiogalactopyranoside at 22 ºC. Bacteria cells were harvest by centrifugation at 4,000 rpm for 30 min at 4 ºC and resuspended in buffer A (25 mM Tris‐HCl, pH 8.0, 200 mM NaCl, 60 mM imidazole). The bacterial suspension was lysed by sonication and centrifuged at 16,000 rpm for 30 min at 4 ºC. The supernatant was applied to a nickel affinity column (Chelating Sepharose Fast Flow, GE Healthcare), and washed with three column volumes of binding buffer to remove the non‐specific proteins. The expressed protein was eluted with buffer B (25 mM Tris‐HCl, pH 8.0, 200 mM NaCl, 250 mM imidazole). The eluted fraction containing the protein was purified by size‐exclusion chromatography (Superdex 200 10/300 GL, GE Healthcare) with buffer C (10 mM Tris‐HCl, pH 8.0, 100 mM NaCl, 5% (v/v) glycerol). The purified PslD was a gift from prof. Lichuan Gu.

### 2.8 Bacterial two‐hybrid system

Bacterial two‐hybrid experiments were conducted as described (Zhang et al., 2009). PCR fragments corresponding to *pslA*, *pslD*, *pslE,* and *pslG* were cloned into the pBT and pTRG vectors. The DNA region containing the signal peptide domain of PslG (PslG_1‐45_) and DNA region without the signal peptide domain of PslG (PslG_31‐442_) were amplified by PCR using genomic DNA isolated from *P. aeruginosa* PAO1. All fusion proteins were confirmed by DNA sequencing. A *hisB* mutant *E. coli* strain XL1‐Blue MRF’ Kan, transformed with the pBT‐ and pTRG‐derived plasmids, was used as reporter strain to screen for positive interactions. Detection of protein‐protein interactions is based on transcriptional activation of the *HIS3* reporter gene, which allows the reporter strain to grow on the M9^+^ His‐dropout Broth (containing 25 μg/ml chloramphenicol and 12.5 μg/ml tetracycline) plate supplemented with 5 mM 3‐amino‐1,2,4‐triazole (3‐AT), a competitive inhibitor of His3 enzyme. pTRG vector carrying *warA* and pBT vector carrying *sadC* fragment were transformed into *E. coli* XL1‐Blue MRF’ Kan and used as a positive control. Positives were verified by using the *aadA* gene, which confers streptomycin resistance, as a second reporter. Cells harboring weaker interactors grew more slowly, requiring longer incubation time for colony development.

### 2.9 Statistical analyses

All the experiments were performed in at least three triplicates. The results are presented as the mean ± *SD*. Student's *t*‐tests were used to evaluate significance.

## 3 RESULTS

### 3.1 PslG and its glycoside hydrolytic activity are involved in the biosynthesis of ePsl in *P. aeruginosa*

**Figure 1:**
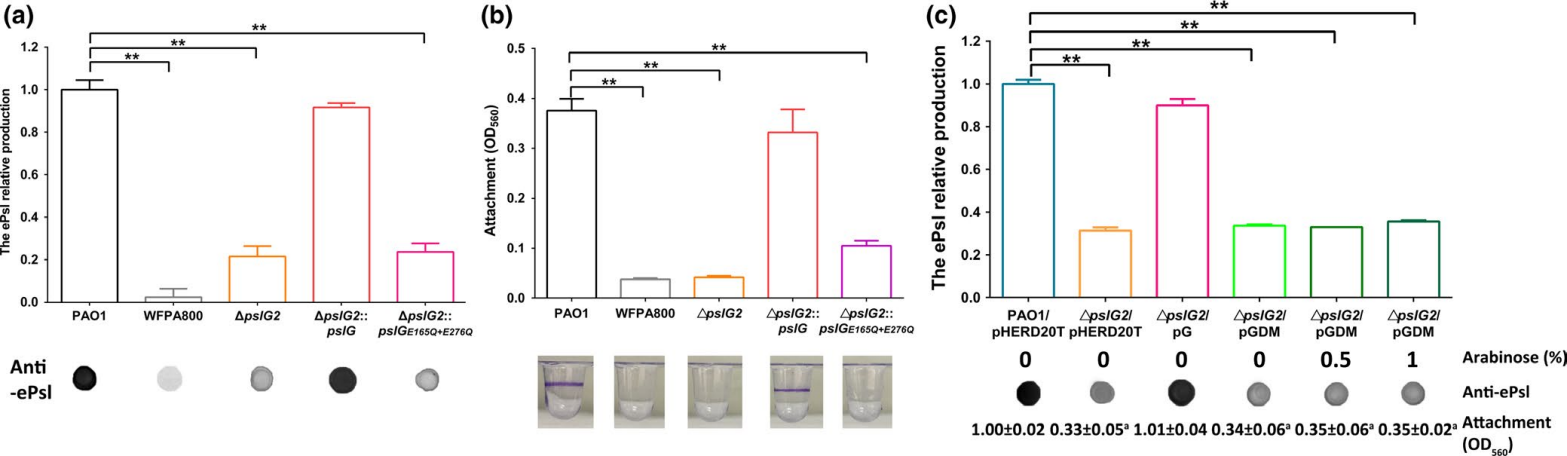
The contribution of PslG and its glycoside hydrolytic activity on the production of ePsl and initial attachment of *P. aeruginosa*. (a) The relative ePsl production of PAO1, ePsl‐negative strain WFPA800, the *pslG* in‐frame deletion mutant Δ*pslG2*, Δ*pslG2*::*pslG*, and the PslG catalytic residues mutant Δ*pslG2*::*pslG_E165Q + E276Q_*. The amount of ePsl is normalized to the level of PAO1. The corresponding anti‐ePsl immune‐dot blot is shown under each bar. (b) Shown is the corresponding initial attachment of the five strains. Values are means from two independent experiments, each with three replicates. The image under each bar is a representative microtiter dish well from corresponding crystal violet biofilm assay. (C) The ePsl production of ∆*pslG2* that complemented by plasmid expressing wild type PslG (pG) or PslG_E165Q + E276Q_ (pGDM). The amount of ePsl is normalized to the level of PAO1/pHERD20T. The corresponding anti‐ePsl immune‐dot blot and arabinose concentration are listed below each bar. The corresponding value of attachment assay for each strain shown under is normalized to the level of PAO1/pHERD20T, the superscript letter “a” indicates a significant difference compared to PAO1/pHERD20T of *p* < 0.01, as determined by Student's *t *test. **p < 0.01, Student's *t* test

Our previous data indicated that overproduced PslG in wild type strain PAO1 reduced the production of ePsl and biofilm biomass, yet overproduced catalytically inactive PslG_E165Q + E276Q_ did not affect the ePsl production and slightly increased biofilm biomass (Yu et al., 2015). These results suggested that PslG might be involved in the biosynthesis of ePsl. To further investigate the role of PslG in ePsl biosynthesis, we constructed an unmarked, non‐polar *pslG* deletion mutant in the PAO1 background named Δ*pslG2*. The immune‐dot blotting showed that the ePsl production of Δ*pslG2* declined up to 80% compared to PAO1 (Figure 1a). We further examined the initial attachment ability of Δ*pslG2* in a microtiter dish because ePsl level impacts bacterial surface‐attachment dramatically. The Δ*pslG2* mutant showed attachment similar to the ePsl‐negative strain WFPA800 (Figure 1b). Flagellum and type IV pili (T4P) also influence the initial attachment of *P. aeruginosa* (Klausen et al., 2003; O'Toole & Kolter, 1998). Therefore, we evaluated the flagellum‐mediated swimming motility and the T4P‐mediated twitching motility, the Δ*pslG2* mutant showed similar levels of swimming and twitching motilities as wild type strain PAO1 (Appendix Figure AA1), indicating the normal function of flagellum and T4P in Δ*pslG2*. The biofilm biomass of Δ*pslG2* was slightly higher than WFPA800 in a 2‐hr biofilm assay (Appendix Figure AA2), indicating the ePsl synthesized from Δ*pslG2* is functional. These results further suggest that PslG is involved in ePsl biosynthesis.

We then further investigated whether the glycoside hydrolytic activity of PslG is important for ePsl production. We constructed a chromosomal site‐mutation strain Δ*pslG2*::*pslG_E165Q + E276Q_* with E165Q and E276Q mutation within PslG. This *pslG* mutant strain showed little ePsl production as that of Δ*pslG2* mutant (Figure 1a). Although the attachment ability of Δ*pslG2*::*pslG_E165Q + E276Q_* was higher than Δ*pslG2*, it was still significantly less than that of PAO1 (fourfold lower than PAO1, Figure 1b). The ePsl production of Δ*pslG2* could be restored by a baseline level expression of PslG (grown without inducer arabinose) from the plasmid pG (PslG was cloned in pHERD20T, Table 1), but it could not be restored by plasmid pGDM (PslG_E165Q + E276Q _in pHERD20T), regardless of the inducer level applied (0%, 0.5%, or 1%) (Figure 1c). The corresponding attachment was also consistent with the ePsl production (Figure 1c, the value shown under each column). These results suggested the importance of PslG glycoside hydrolytic activity in ePsl production and implied that the hydrolytic activity was not only required for degradation of ePsl, but also involved in the biosynthesis of ePsl. Taken together, these results suggested that the PslG and its hydrolytic activity contributed on ePsl production and initial attachment in PAO1.

### 3.2 Inner membrane fraction of PslG is critical for the biosynthesis of ePsl

**Figure 2:**
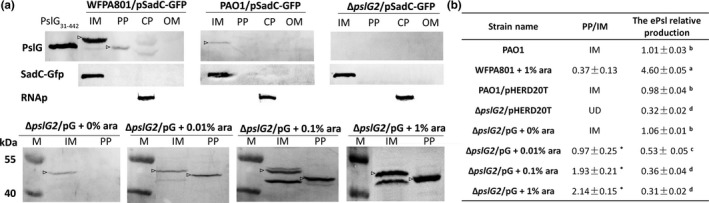
The subcellular localization of PslG and its effect on the biosynthesis of ePsl. (a) Western blotting of the inner membrane (IM), periplasm (PP), cytoplasm (CP), and outer membrane (OM) fractions are shown for PAO1/pSadC‐GFP grown with 1% arabinose, ePsl‐inducible strain WFPA801/pSadC‐GFP grown with 1% arabinose, Δ*pslG*2, and Δ*pslG*2/pG grown with different concentrations of arabinose. Subcellular fractions were probed for PslG, SadC‐Gfp (inner membrane protein, IM), or RNA polymerase (cytoplasmic protein, CP). M: marker. PslG_31‐442_: purified PslG protein loading as the positive control. Arrows indicate protein bands detected by anti‐PslG with right molecular weight. (b) A list of the ratios of PslG localized in periplasm to inner membrane, and the corresponding ePsl production of all tested strains. The amount of ePsl is normalized to the level of PAO1. IM: PslG is mainly detected in the inner membrane. UD: undetectable. Means and *SD* from triplicate experiments are shown. “*” indicates a significant difference compared to WFPA801 of *p* < 0.05, as determined by Student's *t *test. Different superscript letters (a, b, c, d) show significant differences compared to each other at *p* < 0.01, Student's *t *test

The results of Baker et al. (2015) indicated that PslG could localize to both the inner membrane and the periplasm. We further investigated whether the subcellular localization of PslG is important for ePsl biosynthesis. We first detected the localization of PslG in the wild type strain PAO1 by anti‐PslG antibody, PslG was found in the inner membrane fraction, little in the periplasmic fraction (Figure 2a). No band was detected in all fractions from Δ*pslG2* strain (Figure 2a), indicating a PslG‐specific detection. We also determined the PslG localization in the *psl*‐inducible strain WFPA801, which produced high amount of ePsl with arabinose as the inducer. WFPA801 showed a strong PslG band in the inner membrane, a weak band in the periplasmic fraction (3‐fold lower than IM band, Figure 2a) while grown with 1% arabinose. The molecular weight (MW) of protein band detected in the periplasm was similar to the purified protein PslG_31‐442_, indicating that it was a PslG without signal peptide, yet the band detected on inner membrane had a MW of full length PslG. The previous publication showed that SadC was localized in the inner membrane (Zhu et al., 2016). Therefore, we have transferred a plasmid pSadC‐GFP (carrying the *sadC‐gfp* gene, Table 1) into all tested strains in order to use the SadC‐Gfp as a loading control for membrane fraction. In addition, RNA polymerase was used as a loading control for the cytoplasmic fraction. The results of loading controls indicated that the same amount of cell fractions was loaded for each experiment, and each fraction was well separated.

We then further studied whether the expression level of PslG affected its localization. The pG could restore ePsl production of Δ*pslG2* to the level of PAO1 at a baseline level expression of PslG (grown without arabinose) as shown in Figure 1c. While induced with 0.01% and 0.1% arabinose, the ePsl production of Δ*pslG2*/pG was decreased by 47% and 64%, respectively (Figure 2b). A total of 0.1% or 1% arabinose induction decreased the ePsl production of Δ*pslG2*/pG to the level of negative control Δ*pslG2*/pHERD20T (Figure 2b). Accordingly, PslG was detected mainly in the inner membrane fraction of Δ*pslG2*/pG without arabinose (Figure 2a), and the band intensity was similar to that of PAO1. PslG was detected both in the periplasm and inner membrane of Δ*pslG2*/pG inducing with 0.01%, 0.1%, and 1% arabinose (indicated by arrow, Figure 2a). Bands with lower MW in the inner membrane might be partially degraded PslG， which was only found in the PslG‐overexpressed samples (Δ*pslG2*/pG with either 0.1% or 1% arabinose). More PslG was detected in the periplasm of Δ*pslG2*/pG when induced with higher concentration of arabinose (Figure 2a). This suggested that overexpression of PslG led to more PslG releasing to the periplasm. Therefore, we calculated the ratio of PslG in periplasm to inner membrane (Figure 2b, PP/IM). In the ePsl‐inducible strain WFPA801, the transcription of entire *psl* locus was induced by arabinose, its PP/IM value of PslG was 0.37 with 1% arabinose (Figure 2b). For Δ*pslG2*/pG, arabinose only induced the expression of PslG, there was more PslG localized in the periplasm, the PP/IM value of PslG was 0.97, 1.93, and 2.14 while induced with 0.01%, 0.1%, and 1% arabinose, respectively (Figure 2b). These data suggest that some Psl proteins might help PslG stay in the inner membrane. In addition, WFPA801 with 1% arabinose produced large amount of ePsl. However, Δ*pslG2*/pG produced a little ePsl when induced with 0.01%, 0.1%, and 1% arabinose (Figure 2b). The ePsl production of Δ*pslG2*/pG was reduced and the PP/IM value of PslG was elevated while increasing the concentration of arabinose (Figure 2b). These results suggested that PslG localized in the inner membrane was important for the biosynthesis of ePsl and the ratio of PslG in the periplasm to inner membrane determined the amount of ePsl in extracellular.

### 3.3 The localization of PslG is affected by PslA, PslD, and PslE

**Figure 3:**
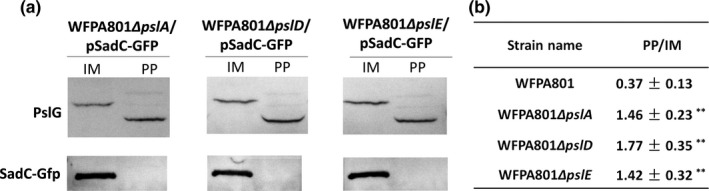
The localization of PslG in the Δ*pslA*, Δ*pslD,* and Δ*pslE* mutants. (a) PslG subcellular localization in *pslA*, *pslD*, and *pslE* in‐frame deletion mutants in the background of WFPA801 containing pSadC‐GFP, respectively. SadC‐Gfp is shown as the inner membrane loading control. (b) The ratio of PslG in periplasm to inner membrane of the three mutants and WFPA801. All strains were grown with 1% arabinose. Means and *SD* from triplicate experiments are shown. “**” indicates a significant difference compared to WFPA801 of *p* < 0.01, as determined by Student's *t *test

To figure out any Psl protein affecting the localization of PslG, we focused on proteins PslA, PslD, and PslE, which were predicted to be localized on the inner membrane and possessed periplasmic domains (Franklin et al., 2011). WFPA801Δ*pslA*, WFPA801Δ*pslD,* and WFPA801Δ*pslE* containing the plasmid pSadC‐GFP were constructed to examine the effect of Psl proteins on the localization of PslG. Western blot results showed that more PslG localized in the periplasm than in the inner membrane in above PslA, PslD, or PslE‐deleted strains (Figure 3a). The ratio of PslG in periplasm to inner membrane was 1.46, 1.77, and 1.42 in PslA, PslD, and PslE mutants (Figure 3b), indicating that these three proteins are important to maintain PslG in the inner membrane.

### 3.4 Protein‐protein interaction among PslE with PslA, PslD, and PslG

We utilized bacterial two‐hybrid system to determine whether there are direct interactions among PslA, PslD, PslE, and PslG (Table 2). pBT and pTRG were empty vectors used as negative control. The interaction of SadC and WarA was used as positive control (McCarthy et al., 2017).The results suggested that there was a direct interaction among PslE with PslG, PslD, or PslA. PslE and PslD showed the strongest interaction (Table 2). We did not detect direct interactions between PslG with either PslA or PslD although they both affected the localization of PslG (Figure 3). These results suggested that PslA and PslD might affect PslG localization through PslE or bacterial two‐hybrid system might not be a best way to detect PslA‐PslG and PslD‐PslG interactions.

**Table 2:** Protein interactions among PslA, PslD, PslG, and PslE

a *E. coli* strain containing		pBT							
		SadC	none	PslA	PslG	PslG_1−45_	PslG_3__1−442_	PslE	PslD
pTRG	WarA	**+**	ND	ND	ND	ND	ND	ND	ND
	none	ND	**‐**	**‐**	**‐**	**‐**	**‐**	**‐**	**‐**
	PslA	ND	**‐**	**‐**	**‐**	**‐**	**‐**	**++**	**‐**
	PslG	ND	**‐**	**‐**	**‐**	**‐**	**‐**	**+**	**‐**
	PslG_1−45_	ND	**‐**	**‐**	**‐**	**‐**	**‐**	**‐**	**‐**
	PslG_3__1−442_	ND	**‐**	**‐**	**‐**	**‐**	**‐**	**‐**	**‐**
	PslD	ND	**‐**	**‐**	**‐**	**‐**	**‐**	**+++**	**‐**
	PslE	ND	**‐**	**‐**	**‐**	**‐**	**‐**	**‐**	**+**

a Proteins expressed from bait vector pBT were listed in a row, proteins expressed from target vector pTRG were listed in a vertical column. The interaction of SadC and WarA was used as positive control. Protein interactions in the *E. coli* strain XL1‐Blue MRF’ Kan were detected by the ability of the cells to grow on the M9^+^ His‐dropout Broth plate supplemented with 5 mM 3‐AT and 12.5 μg/ml streptomycin. The strength of interaction was based on the growth rate of cells on the plate. ‐: no interaction. +: weak interaction. ++: moderate interaction. +++: strong interaction. ND: not determined.

To know whether the full length of PslG is necessary for the interaction with other Psl proteins, we detected the interaction of the N‐terminal 45 amino acid residues of PslG (PslG_1‐45_, contained the entire signal peptide domain) or PslG_31‐442_ (contained only the soluble domain of PslG) with PslA, PslD, or PslE (Table 2). No interactions were found for either PslG_1‐45_ or PslG_31‐442_ with these three Psl proteins. These results suggested that the interaction with PslE required a full length PslG.

**Figure 4:**
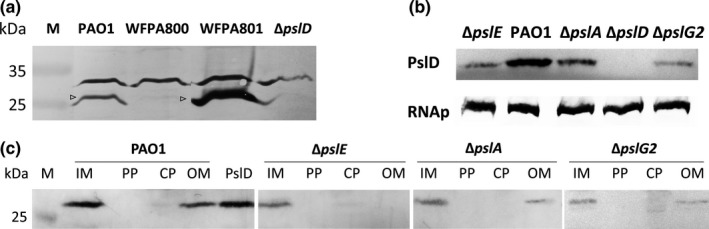
The effect of PslE on the localization of PslD. (a) Western blotting analysis of whole cell extracts of PAO1, WFPA800, WFPA801 grown with 1% arabinose, and Δ*pslD* using anti‐PslD antibody. The molecular weight of PslD is 27.9 kDa. Arrows indicate the bands of PslD protein. (b) Western blotting analysis of whole cell extracts of Δ*pslE*, PAO1, Δ*pslA*, Δ*pslD,* and Δ*pslG2* using anti‐PslD and anti‐RNA polymerase (RNAp) antibody, respectively. (C) Identification of PslD in the IM, PP, CP, and OM fractions from PAO1 and its derived Δ*pslA*, Δ*pslE,* and Δ*pslG2* mutants by western blotting analysis with anti‐PslD antibody. PslD: purified PslD protein loading as the positive control. M: marker

PslE‐PslD showed the strongest interaction, thus we further asked whether PslE can affect the localization of PslD. To detect PslD, we made an anti‐PslD antibody by an antigenic epitope approach. This antibody was first examined for its specificity by western blotting against the whole cell extracts of PAO1 and WFPA801 as positive controls, WFPA800 and Δ*pslD* strain as negative controls (Figure 4a, arrows indicated the bands of PslD protein). Then this anti‐PslD antibody was used for the detection of PslD. We first examined the PslD in the whole cell extract from PAO1, Δ*pslE*, Δ*pslA,* Δ*pslD,* and Δ*pslG2* strains. RNA polymerase was used as a loading control (Figure 4b). The whole cell extract of wild type PAO1 had more PslD than that of the three mutant strains (Figure 4b). We then investigate PslD's localization. PslD was found to be enriched in both the inner membrane fraction and outer membrane fraction in PAO1 (Figure 4c). Then we extracted membrane fractions of Δ*pslE*, Δ*pslA,* and Δ*pslG2*. PslD was detected only in the inner membrane but not in outer membrane in the absence of PslE (Figure 4c), while deletion of *pslA* or *pslG* had no influence on PslD localization (Figure 4c). These results were consistent with the results of proteins interactions (Table 2), suggesting that PslE might help PslD to span to the outer membrane by direct PslE‐PslD interaction.

## 4 DISCUSSION

The ePsl is a key biofilm matrix component of the life‐threaten pathogen *P. aeruginosa*. It promotes bacteria cell‐cell and cell‐surface interaction by acting as a “molecular glue” (Ma et al., 2009, 2006); it forms a fiber‐like matrix to protect bacteria from antibiotics and phagocytic cells (Billings et al., 2013; Mishra et al., 2012); and it can function as a signal to stimulate biofilm formation (Irie et al., 2012). However, the molecular mechanism of ePsl biosynthesis remains unknown. In this study, we focused on the role of glycoside hydrolase PslG in the biosynthesis of ePsl. We investigated the protein interactions of PslA, PslD, PslE, and PslG and examined the effects of protein interactions on protein localization of PslD and PslG. Our data suggested that the membrane‐associated PslG was a part of ePsl biosynthesis machinery and the Psl proteins interactions might control the release of PslG into the periplasmic space.

Glycoside hydrolases are common in many bacterial exopolysaccharide biosynthesis operons, such as PssZ in *Listeria monocytogenes* (Koseoglu et al., 2015), PgaB and BcsZ in *E. coli* (Mazur & Zimmer, 2011; Wang, Preston, & Romeo, 2004), and WssD and AlgL in *Pseudomonas fluorescence* (Bakkevig et al., 2005; Spiers, Bohannon, Gehrig, & Rainey, 2003). Our previous study demonstrated the structure of glycoside hydrolase PslG and its effects on biofilm when applied exogenously (Yu et al., 2015), while little is known about its function in the process of ePsl biosynthesis. Baker et al. (2015) had studied the role of *pslG* in a *psl* overexpression strain PAO1Δ*pelF*P_BAD_ *psl*. They concluded that *pslG* had no involvement in the biosynthesis of ePsl. However, in a *psl* overexpression system, only a huge change on ePsl production could be find. Therefore, to determine the role of PslG and its endoglycosidase activity in the biosynthesis of ePsl in *P. aeruginosa* PAO1, we constructed strain Δ*pslG2* and Δ*pslG2*::*pslG_E165Q + E276Q_*, and found that PslG and its hydrolytic activity were important for initial attachment and ePsl production. Monday and Schiller (1996) and Penaloza Vazquez, (1997) considered AlgL functions as the integral component in the alginate biosynthesis complex and lacking of *algL* resulted in less alginate production. Here lacking of *pslG* decreased ePsl production, suggesting PslG serves as the integral component in the ePsl biosynthesis complex. The ePsl production of Δ*pslG2* could not be restored by PslG_E165Q + E276Q_, indicating the hydrolytic activity of PslG is critical for optimal ePsl biosynthesis, similar to the cellulose degrading enzyme, BcsZ (Mazur & Zimmer, 2011). Though the differences in ePsl production between WFPA800, Δ*pslG2*, and Δ*pslG2*::*pslG_E165Q + E276Q_* were not enough to make significant differences in a 30 min attachment assay, the differences of biofilm biomass could be found in a biofilm assay post 2 hr incubation (Appendix Figure AA2), in which the biofilm biomass of Δ*pslG2*, and Δ*pslG2*::*pslG_E165Q + E276Q_* were slightly higher than WFPA800, suggesting the ePsl synthesized from *pslG* mutants is functional.

PslG localizes in the inner membrane and periplasm (Baker et al., 2015). We are interested in whether the specific localization of PslG plays different role in the biosynthesis of ePsl. We found PslG in wild type PAO1 mainly localized in the inner membrane. When PslG was overexpressed alone, more PslG localized in the periplasm with a decrease in ePsl production. These results suggest inner membrane association of PslG helps synthesize ePsl polymer, while PslG in the periplasm may degrade ePsl polymer randomly.

**Figure 5:**
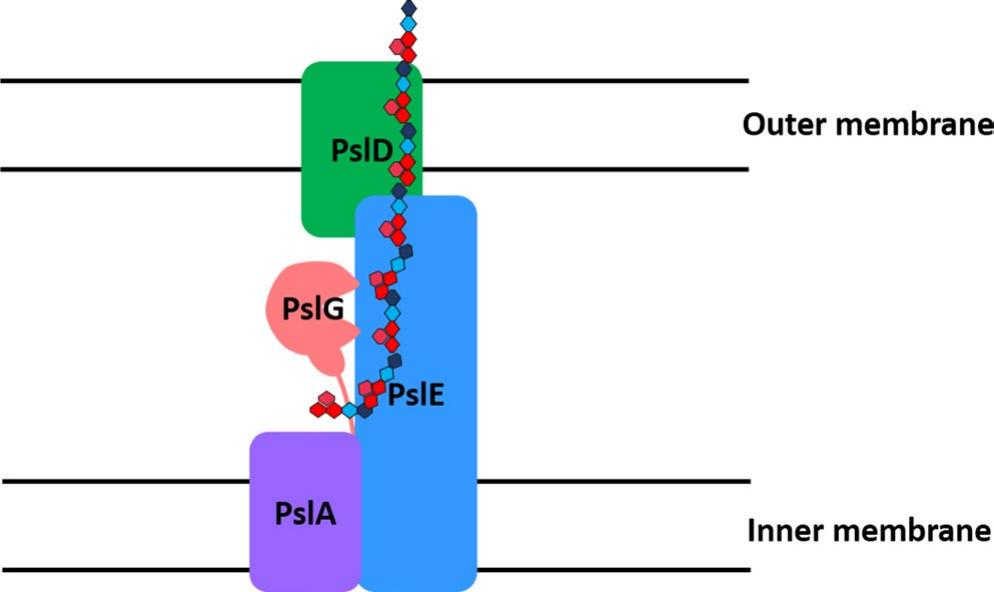
A schematic view of Psl proteins in the ePsl biosynthesis machinery. Membrane associated PslG is a part of the ePsl biosynthesis machinery, in which PslA, PslD, and PslE allow PslG in an optimal localization to control the degradation of ePsl polymer at certain length. PslE interacts with PslA, PslD, and PslG, it helps PslD to localize in the outer membrane to export the ePsl

As the localization of PslG is critical to ePsl production, we have further investigated other Psl proteins that might modulate the localization of PslG. We focus on the predicted periplasmic proteins (PslA, PslD, and PslE) that may interact with PslG in the ePsl assembly apparatus. We found that more PslG localized to the periplasm in the absence of PslA, PslD, or PslE. Interaction of PslE with PslG was further confirmed via bacterial two‐hybrid assay. These results suggested that the membrane‐associated PslG was a part of ePsl biosynthesis machinery, in which PslA, PslD, and PslE might help control or delay the release of PslG into periplasmic space. Our data have also shown that the hydrolytic activity of PslG is important for the synthesis of ePsl, implying that the ePsl biosynthesis machinery may allow PslG in an optimal localization to control the degradation of ePsl polymer at certain length (Figure 5).

The structures and functions of PslA, PslD, and PslE have not been experimentally determined. PslA might likely play a similar role to WbaP in providing a site for the assembly of the oligosaccharide repeating unit onto the isoprenoid lipid at the cytoplasmic face of the inner membrane (Franklin et al., 2011; Whitfield, 2006). PslE has characteristic domains of a Wzz (or Wzc) homolog and is therefore predicted to act as the polysaccharide co‐polymerase (PCP) component in this system (Franklin et al., 2011; Larue, Kimber, Ford, & Whitfield, 2009). The periplasmic domain of PCPs is proposed to affect polysaccharide chain length (Tocilj et al., 2008) and is thought to form critical interactions with the CPS/EPS export component thereby completing a complex that facilitates transfer of the polymer through the periplasm (Cuthbertson, Mainprize, Naismith, & Whitfield, 2009). PslD is predicted to be the polysaccharide exporter with structural similarity to the *E. coli* K30 capsule translocase, Wza, an integral outer membrane lipoprotein (Dong et al., 2006; Franklin et al., 2011). Predicted PslD 3‐dimensional structure (Appendix Figure AA3) has indicated that most of PslD can be structurally modeled onto Wza (PDB ID 2J58), but there is a clear difference, PslD appears to lack the outer membrane barrel and large periplasmic domain. Therefore, it is difficult to understand how the Psl polymer is translocated across the outer membrane. In this study, we found that PslD had a strong interaction with PslE and it could not localize to the outer membrane without PslE, which suggest that PslE, the Wzc homolog, interacts with PslD and helps PslD localize to the outer membrane. In addition, our data also suggest PslE is likely to act as the periplasmic scaffold and recruit proteins to form a polysaccharide biosynthetic complex because PslE can interact with PslA, PslD, and PslG (Table 2, Figure 5). More PslD was detected in PAO1 than in *pslA*, *pslE*, or *pslG* deletion mutant, implying that PslD integrated into the ePsl biosynthetic complex is more stable than free PslD.

To the best of our knowledge, this is the first study to investigate the connection between protein interactions and their localizations during ePsl biosynthesis of *P. aeruginosa.* Our data showed the glycoside hydrolase PslG and its hydrolytic activity were important to ePsl production of *P. aeruginosa*. The inner membrane association of PslG might be involved in the biosynthesis of ePsl, while PslG localized in the periplasm may degrade ePsl. We have experimentally proved the PslE interacted with PslA, PslD, and PslG in vivo. All the three proteins, PslA, PslD, and PslE, had an impact on PslG localization, which was critical to ePsl biosynthesis. PslE helped PslD localize the outer membrane, these two proteins might form a complex to help transport Psl across the outer membrane. In summary, we have shown in this study that ePsl biosynthesis is a complex processing with dynamic protein‐protein interactions, leading to the assembly of ePsl biosynthesis machinery.

## CONFLICT OF INTERESTS

The authors declare that there is no conflict of interest.

## AUTHOR CONTRIBUTIONS

H.W., D.W., and L.Z.M. conceived and designed experiments, and contributed to the writing of the manuscript. H.W. and M.T. conducted experiments.

## ETHICS STATEMENT

None required.

## Figures and Tables

**Figure A1:**
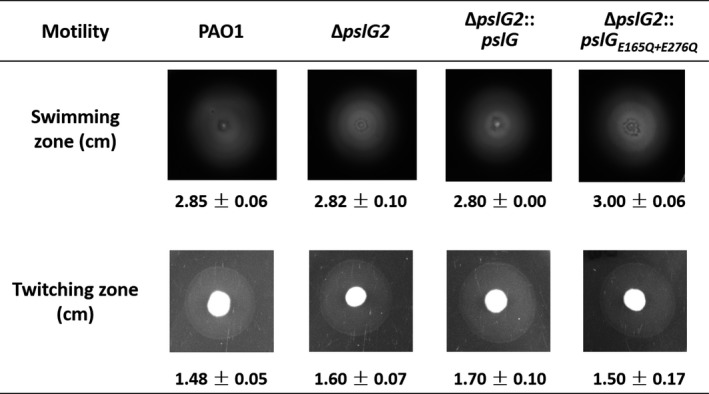
The flagella mediated swimming motility and type IV pili mediated twitching motility were tested for PAO1 and its derived *pslG* mutants. Diameters of the zones were averaged from triplicate experiments

**Figure A2:**
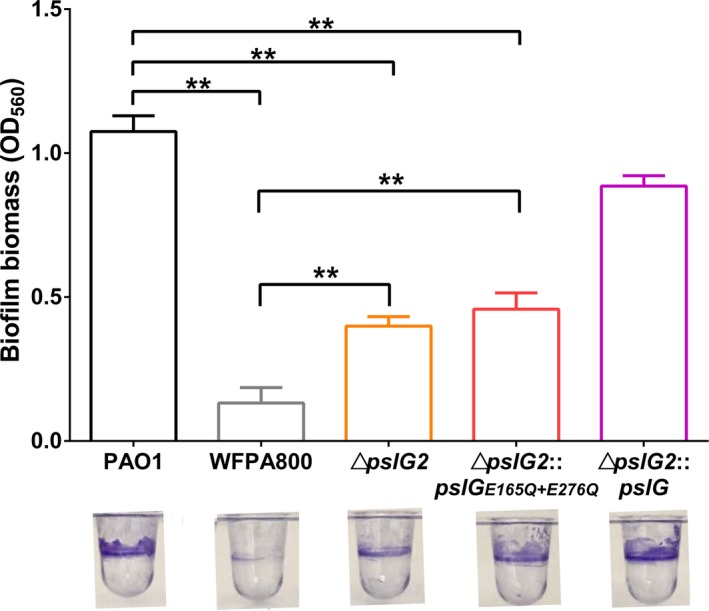
The results of 2‐hr biofilm assay of PAO1, WFPA800, Δ*pslG2*, Δ*pslG2*::*pslG*, and Δ*pslG2*::*pslG_E165Q + E276Q_*. Means and *SD* from triplicate repeats are shown. **p < 0.01, Student's *t *test

**Figure A3:**
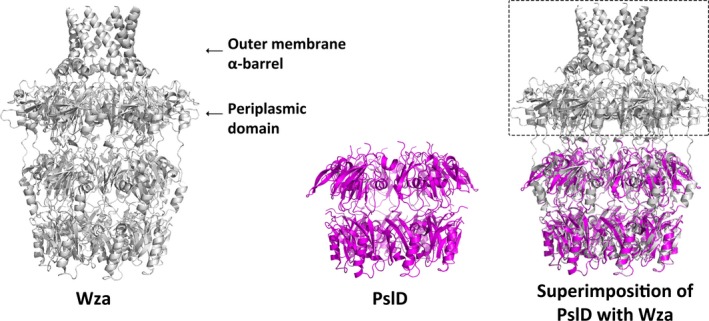
The schematic view of predicted PslD 3‐D structure and its comparison with Wza (PDB ID 2J58). Structures are shown in cartoon representation. The structure of Wza is shown in gray, and predicted PslD is shown in magenta, respectively. Dotted box indicates the domain lacking in the structure of PslD

## Data Availability

All data are provided in full in this paper.
